# Metabolic transition in mycorrhizal tomato roots

**DOI:** 10.3389/fmicb.2015.00598

**Published:** 2015-06-23

**Authors:** Javier Rivero, Jordi Gamir, Ricardo Aroca, María J. Pozo, Víctor Flors

**Affiliations:** ^1^Department of Soil Microbiology and Symbiotic Systems, Estación Experimental del Zaidín – Consejo Superior de Investigaciones CientíficasGranada, Spain; ^2^Metabolic Integration and Cell Signaling Laboratory, Associated Unit UJI-CSIC, Plant Physiology Section, Department of Ciencias Agrarias y del Medio Natural, Universitat Jaume ICastellón, Spain; ^3^Unit of Plant Biology, Department of Biology, University of FribourgFribourg, Switzerland

**Keywords:** arbuscular mycorrhiza, metabolomics, *Funneliformis mosseae*, *Rhizophagus irregularis*, oxylipins

## Abstract

Beneficial plant–microorganism interactions are widespread in nature. Among them, the symbiosis between plant roots and arbuscular mycorrhizal fungi (AMF) is of major importance, commonly improving host nutrition and tolerance against environmental and biotic challenges. Metabolic changes were observed in a well-established symbiosis between tomato and two common AMF: *Rhizophagus irregularis* and *Funneliformis mosseae*. Principal component analysis of metabolites, determined by non-targeted liquid chromatography–mass spectrometry, showed a strong metabolic rearrangement in mycorrhizal roots. There was generally a negative impact of mycorrhizal symbiosis on amino acid content, mainly on those involved in the biosynthesis of phenylpropanoids. On the other hand, many intermediaries in amino acid and sugar metabolism and the oxylipin pathway were among the compounds accumulating more in mycorrhizal roots. The metabolic reprogramming also affected other pathways in the secondary metabolism, mainly phenyl alcohols (lignins and lignans) and vitamins. The results showed that source metabolites of these pathways decreased in mycorrhizal roots, whilst the products derived from α-linolenic and amino acids presented higher concentrations in AMF-colonized roots. Mycorrhization therefore increased the flux into those pathways. Venn-diagram analysis showed that there are many induced signals shared by both mycorrhizal interactions, pointing to general mycorrhiza-associated changes in the tomato metabolome. Moreover, fungus-specific fingerprints were also found, suggesting that specific molecular alterations may underlie the reported functional diversity of the symbiosis. Since most positively regulated pathways were related to stress response mechanisms, their potential contribution to improved host stress tolerance is discussed.

## Introduction

Beneficial organisms are common in the rhizosphere and they provide important ecosystem services (Philippot et al., 2013). They can greatly contribute to plant performance by improving nutrition, stress tolerance and plant phenotypic plasticity, an important advantage in heterogeneous environments where precise allocation of limited resources between growth and stress resistance is critical for survival (Pozo et al., 2015). Among these beneficial organisms, soil-borne fungi from the phylum Glomeromycota, known as arbuscular mycorrhizal fungi (AMF) deserve special attention. They are able to establish the most ancient and widespread plant–fungal symbiosis, known as arbuscular mycorrhizas (AM), with more than 80% of all terrestrial plant species (Smith and Read, 2008). AMF are obligate biotrophs, and it is assumed that the host plant allocates photosynthates to the fungus for the formation, maintenance, and function of mycorrhizal structures (Bago et al., 2000). In return, the AMF improve plant acquisition of water and mineral nutrients. This symbiosis is extremely important for the uptake of inorganic phosphate, but also contributes to the uptake of nitrogen (N) and various trace elements (Smith and Smith, 2011; Hodge and Storer, 2015). Besides plant nutrition, the symbiosis impacts the plant’s ability to overcome biotic and abiotic stresses, commonly improving host tolerance to unfavorable environmental conditions and resistance to pathogens (Gianinazzi et al., 2010; Jung et al., 2012; Ruíz-Lozano et al., 2012; Selosse et al., 2014). The establishment and maintenance of the association requires a high degree of coordination between both partners, and bidirectional (plant and fungal) control assures a fair trade of resources between the symbionts (Kiers et al., 2011). Indeed, a precise regulation of host hormone levels has been proposed as a central mechanism in the regulation of the interaction (Pozo et al., 2015).

Although there is no strict partner specificity in AM, the outcome of AM interactions depends on the interacting partners and the environmental conditions (Walder et al., 2012; Smith and Smith, 2015). Actually, there is evidence for “functional diversity” occurring as plant and fungal genotypes determine the benefits of the interaction; some combinations being more efficient than others in terms of nutrition and/or stress resistance (Feddermann et al., 2010; Jung et al., 2012; Mensah et al., 2015; Smith and Smith, 2015). For example, colonization of roots by *Funneliformis mosseae* or *Rhizophagus irregularis*, the two widespread AMF used in this study, resulted in different levels of bioprotection against Phytophthora root rot in tomato or Fusarium wilt in melon, and to drought stress in lettuce (Pozo et al., 2002; Marulanda et al., 2003; Porcel et al., 2006; Martínez-Medina et al., 2009).

Different studies have tried to unravel the molecular mechanisms regulating AM and their impact on plant fitness, most of them focused on differential gene expression and protein profiles (Liu et al., 2007; Guether et al., 2009; López-Ráez et al., 2010; Abdallah et al., 2014). A consistent output of “omic” studies on AM has been the observation that mycorrhizal colonization significantly impacts host gene expression and metabolomic profiles (Salvioli and Bonfante, 2013). Metabolomics is a valuable technology which provides comprehensive quantitative profiling of metabolites in biological systems; and liquid or gas chromatography coupled with mass spectrometry (LC–MS or GC–MS) are widely used analytical tools for such untargeted metabolomic studies (De Vos et al., 2007). The first metabolomic studies in AM interactions were targeted analyses focusing on a few well-characterized metabolites to monitor plant responses (Stumpe et al., 2005; Sawada et al., 2009; López-Ráez et al., 2010), however, a whole picture of the impact of AM on the general metabolic profile was missing. Nowadays, metabolomic untargeted approaches allow the separation and detection of a wide range of metabolites, such as amino acids, fatty acids, organic acids, sugar phosphates, nucleotides, and glycoside derivatives, providing a global fingerprint about the quantitative and qualitative changes in the secondary metabolism of the host plant (De Vos et al., 2007). The LC–MS technique is highly sensitive for the detection of key molecules in the phenotypic mechanisms underlying organism responses to abiotic or biotic interactions (Sardans et al., 2011; Gamir et al., 2012). Indeed, untargeted metabolomic studies have revealed the importance of metabolic reprogramming as a determinant in other plant–microbe symbioses, e.g., legume–rhizobia associations (Zhang et al., 2012) and ectomycorrhizas in poplar (Tschaplinski et al., 2014).

Few studies have addressed the metabolome reprogramming associated with AM and knowledge concerning metabolomic transitions in mycorrhizal plants remains restricted to a few recent studies. Concerning changes in mycorrhizal roots, research has been limited to legumes, in particular to the model plant *Medicago truncatula* and to one AMF strain, *R. irregularis* (Schliemann et al., 2008; Laparre et al., 2014). The studies have revealed an impact of *R. irregularis* colonization on primary and secondary metabolism, mainly on amino acids (glutamic acid, aspartic acid, and asparagine), fatty acids (palmitic and oleic acids), apocarotenoids (cyclohexenone and mycorradicin derivatives), and isoflavonoids (daidzein, ononin, and malonylononin). Remarkably, the impact of the symbiosis on the host metabolism extends to aboveground tissues and can vary with environmental conditions (Fester et al., 2011). Moreover, recent multispecies metabolomic analysis of leaves from *R. irregularis*-colonized plants showed a common core of mycorrhiza-related and highly accumulated metabolites shared by dicotyledonous and monocotyledonous plants, although they also evidenced many species-specific responses (Schweiger et al., 2014).

This study aims to decipher the impact of a well-established mycorrhizal association with two widespread and well characterized AMF (*F. mosseae* and *R. irregularis*) in the root metabolic profile of a non-legume, tomato (*Solanum lycopersicum*), where the benefits of AM have been shown to be agronomically relevant in terms of stress resistance and fruit quality (Pozo et al., 2002; Fritz et al., 2006; Aroca et al., 2008; Gianinazzi et al., 2010; Barzana et al., 2012; Giovannetti et al., 2012; Zouari et al., 2014). LC–MS revealed important changes in the metabolome of mycorrhizal tomato roots and we discuss the potential relevance of these changes in host fitness.

## Materials and Methods

### Plant Material and AMF Inoculation

Arbuscular mycorrhizal fungi isolates of *R. irregularis* (BEG 121; formerly *Glomus intraradices*) and *F. mosseae* (BEG12; formerly *Glomus mosseae*) from the International Bank of Glomeromycota^1^ are continuously maintained in an open-pot culture of *Trifolium repens* L. mixed with *Sorghum vulgare* Pers. (Steud.) Millsp. & Chase plants in a greenhouse. The inocula consist of substrate (vermiculite/sepiolite, 1:1), spores, mycelia, and infected root fragments from those cultures. Tomato seeds (*Solanum lycopersicum* L. cv. Moneymaker) were surface disinfected by immersion in 4% NaHClO (10 min) containing 0.02% (v/v) Tween 20^®^, rinsed thoroughly with sterile water and incubated for 3 days in an open container with sterile vermiculite at 25°C in darkness. Plantlets were transferred to 1 L pots containing a sterile sand:soil (1:1) mixture. Pots for mycorrhizal treatments were inoculated by adding 10% (v/v) *F. mosseae* or *R. intraradices* inoculum. Uninoculated control plants received the same amount of autoclaved mycorrhizal inoculum together with a 3 ml aliquot of a filtrate (<20 μm) of both AM inocula, in order to provide the general microbial population but free of AMF propagules.

A total of ten plants were used for each treatment. Plants were randomly distributed and grown in a greenhouse at 24/16°C with a 16/8 h diurnal photoperiod and 70% humidity. Plants were watered three times a week with Long Ashton nutrient solution (Hewitt, 1966) containing 25% of the standard phosphorus (P) concentration, and water was supplied daily to maintain the substrate at 100% field capacity, as reported in El-Mesbahi et al. (2012). Plants were harvested after 8 weeks, the fresh weight of shoots and roots was determined, and the material immediately frozen in liquid N and stored at -80°C. An aliquot of each individual root system was reserved for mycorrhizal quantification.

### Determination of Mycorrhizal Colonization

Mycorrhizal colonization was estimated after clearing washed roots in 10% KOH and subsequent staining of fungal structures with 5% ink in 2% acetic acid (Vierheilig et al., 2005). The extent of mycorrhizal colonization (expressed as percentage of total root length colonized by the AMF) was calculated according to the gridline intersection method (Giovannetti and Mosse, 1980) using a Nikon Eclipse 50i microscope and bright field conditions.

### Phosphorus, Carbon, and Nitrogen Content

Total P, carbon (C), and N content in the roots was measured at the Ionomic Laboratory of Technical Services of the *Centro de Edafología y Biología Agraria del Segura* (CSIC), Murcia, Spain. Three biological replicates, each consisting of a pool of roots from three independent plants (nine plants in total), were analyzed for each treatment. Frozen roots were ground to a fine powder and lyophilized. P concentrations were analyzed after an acid digestion of the samples, by inductively coupled plasma optical emission spectrometry (ICP-OES; ICAP 6500 DUO THERMO). Total C and N contents were determined using an Elemental Analyzer (LECO TRUSPEC CN) according to standard procedures.

### Analysis of Gene Expression by RT-qPCR

Total RNA from tomato roots was extracted and treated with DNase using the Direct-zol RNA MiniPrep kit (Zymo Research). Subsequently, the RNA was purified through a column using the RNA Clean & Concentrator-5 kit (Zymo Research), and stored at -80°C until use. The first-strand cDNA was synthesized with 1 μg of purified total RNA using the iScript cDNA Synthesis kit (Bio-Rad). Four independent biological replicates were analyzed per treatment. All kits were used according to the manufacturer’s suggested protocols.

Relative quantification of specific mRNA levels was performed using the comparative 2^-Δ(ΔCt)^ method (Livak and Schmittgen, 2001). Expression values were normalized using the housekeeping gene *SlEF-1*α (López-Ráez et al., 2010), which encodes for the tomato elongation factor-1α. The functionality of AM symbiosis was quantified using the marker gene *LePT4*, which encodes a mycorrhiza-inducible phosphate transporter expressed in arbusculated cells (Balestrini et al., 2007). Nucleotide sequences of the primers used were: *SlEF-1*α-F 5′-GATTGGTGGTATTGGAACTGTC-3′, *SlEF-1*α-R 5′-AGCTTCGTGGTGCATCTC-3′; *LePT4*-F 5′-GAAGGGGAGCCATTTAATGTGG-3′, *LePT4*-R 5′-ATCGCGGCTTGTTTAGCATTTC-3′.

### Reagents and Standards

All standards, including amino acids, salicylic acid, phenols, IAA, 5-Hydroxyindole-3-acetic acid, Indole-3-acetamide, *N*-(3-indoley lacetyl)-L-alanine, Indole-3-carboxaldehyde, Methyl indole-acetate, jasmonic acid (JA), abscisic acid, salicylic acid glucoside ester, OPDA, carboxylic acids, and sugars were purchased from SIGMA (Barcelona, Spain). Methanol (HPLC grade) was obtained in SIGMA (Barcelona, Spain), formic acid and NaOH were obtained from J.T Baker (Deventer, Holland). Indole-3-carboxylic acid and 1,4-diaminobutane were obtained from VWR (Barcelona, Spain).

### Liquid Chromatography and ESI Mass Spectrometry

#### LC–ESI Full Scan Mass Spectrometry (Q-TOF Instrument)

Freeze-dried roots (50 mg) were homogenized on ice in 1 ml of MeOH:H2O (10:90) containing 0.01% of HCOOH. The homogenate was centrifuged at 15000 rpm for 15 min at 4°C. The supernatant was recovered and filtered through 0.2 μm cellulose filters (Regenerated Cellulose Filter, 0.20 μm, 13 mm D. pk/100; Teknokroma). A 20 μl aliquot of was injected in the HPLC. The full metabolomic profiling was performed using an Acquity UPLC system (Waters, Mildford, MA, USA) interfaced to hybrid quadrupole time-of-flight (QTOF MS Premier). Analytes were eluted with an aqueous methanol gradient containing 0.01% HCOOH. Three biological replicates, each consisting of a pool of three independent plants (nine plants in total), were randomly injected in duplicate for every treatment. The LC separation was performed with an HPLC SunFire C18 analytical column, 5 μm particle size, 2.1 mm × 100 mm (Waters). Solvent gradients and further chromatographic conditions were performed as previously described (Gamir et al., 2012; Agut et al., 2014). The LC–ESI Q-TOF MS library of plant compounds was used for a straight identification in full-scan analysis. Standards for phenols, indolic compounds, amino acids, hormones and their derivatives (up to 93 compounds) were prepared (100 ppb) in a composite solution (Supplementary Table S2). The standard solution was injected through the HPLC in both positive and negative electro-spray ionization (ESI+; ESI-) to identify compounds by matching exact mass and retention time between standard and experimental samples.

#### Full Scan Data Analysis

Data were acquired in centroid mode and subsequently transformed into cdf files using the Databridge from MassLynx 4.1 software (MassLynx 4.1, Waters). Chromatographic signals were processed using the software R for statistical purposes^2^. Signals from ESI+ and ESI- were processed separately. Peak peaking, grouping and signal corrections were performed using the XCMS algorithm (Smith et al., 2006). Metabolite amounts were analyzed on the basis of normalized peak area units relative to the dry weight. The Kruskal–Wallis test (*p* < 0.05) was applied to analyze the metabolomic differences between treatments. To determine a global behavior of the signals, principal component analyses (PCA) plots were generated using the Multibase 2015 algorithm^3^. Statistical and heat map analysis were performed using the MarVis Suit 2.0 software tool for clustering and visualization of metabolic biomarkers (Kaever et al., 2014). Adduct, isotope correction, clustering, and color heat map visualization were also performed by using associated software packages MarVis Filter and MarVis Cluster.

### Statistical Analyses

All statistical analyses (ANOVA, *post hoc*, and *t*-test) were conducted using Statgraphics Plus 3.1 (Rockville, MD, USA), “R” software version 2.9.2 (R Development Core Team)^2^ and the XCMS package.

## Results

### Root Colonization by *F. mosseae* and *R. irregularis* and Physiological Status of the Plant

**FIGURE 1:**
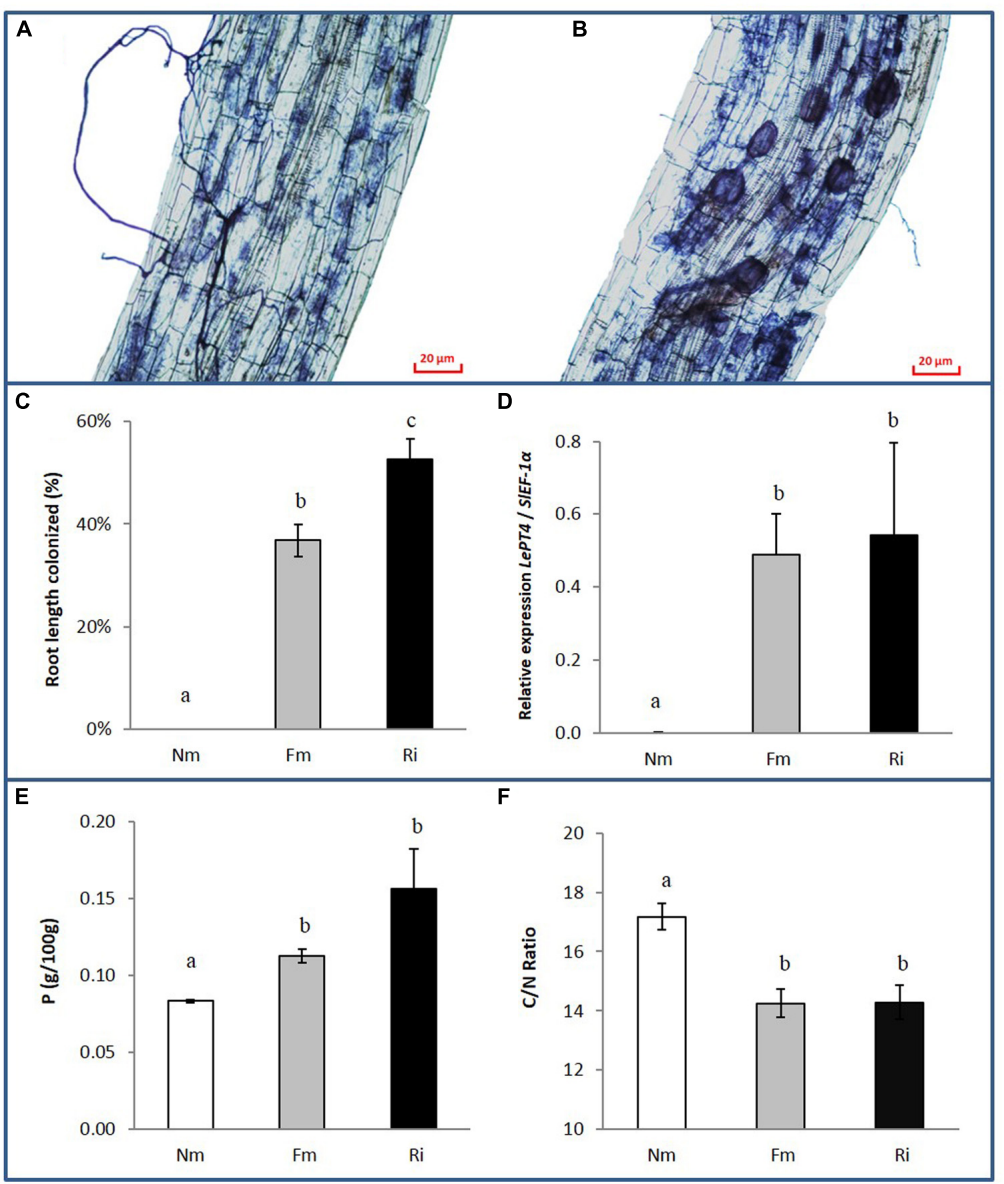
**Fungal colonization and nutrient content in non-mycorrhizal (Nm) and mycorrhizal tomato roots colonized by either *Funneliformis mosseae* (Fm) or *Rhizophagus irregularis* (Ri), 8 weeks after inoculation. (A)** Ink-staining of fungal structures in Fm and **(B)** Ri colonized roots. **(C)** Percentage of root length colonized by the mycorrhizal fungi. Data represent the means of 10 independent biological replicates ± SE. **(D)** Expression levels of the tomato gene *LePT4* normalized to the housekeeping tomato gene *SlEF-1*α. Data represent the means of four independent biological replicates ± SE. **(E)** Root P content and **(F)** C/N root content ratio. Data represent the means of three independent replicates each consisting of a pool of roots from three independent biological replicates ± SE. Data not sharing a common letter differ significantly (*p* < 0.05) according to the Newman–Keuls test.

Plants were harvested 8 weeks after inoculation with the mycorrhizal fungi. Root and shoot fresh weights were determined and mycorrhizal colonization, P, C, and N content in the roots were analyzed (**Figure 1** and Supplementary Table S1). Staining of fungal structures within the roots showed that the mycorrhizal symbiosis was well established in both inoculation treatments, with abundant fungal colonization of the root cortex and well-formed arbuscules. Vesicles, the fungal storage structures, were more abundant in the roots with the most effective colonizer (*R. irregularis*), as has been described in previous studies (López-Ráez et al., 2010; **Figures 1A,B**). Absence of fungal structures was confirmed in roots of the non-mycorrhizal controls, and the extent of root length colonized by *F. mosseae* or *R. irregularis* differed significantly (**Figure 1C**, *p* < 0.01). The functionality of the symbiosis was assessed by analyzing the expression of the tomato gene *LePT4*, which encodes a phosphate transporter induced in arbuscule containing cells, where most of the nutrients exchange takes place; it is therefore used as marker of a functional symbiosis. A very strong induction of *LePT4* expression was detected in mycorrhizal roots, reaching similar levels in the interaction with both fungi (**Figure 1D**).

The symbiosis did not have a significant effect on shoot or root biomass under our experimental conditions (Supplementary Table S1). However, both AM treatments enhanced the P content in roots compared to those from non-mycorrhizal controls (**Figure 1E**). Similarly, N content was higher in mycorrhizal roots (Supplementary Table S1), while total carbon content in the roots remained unaltered. Accordingly the C/N ratio showed a significant reduction in mycorrhizal roots (**Figure 1F**).

### AMF Colonization has a Strong Impact on the Metabolic Profile of the Host Roots

We analyzed the reprogramming of the tomato root metabolism associated with well-established symbiosis with each AMF. Following the chromatographic analysis, a bioinformatic processing of the detected signals was performed, and cluster and functional pathway analyses were performed in order to obtain plausible biological information of such metabolic reprogramming.

**FIGURE 2:**
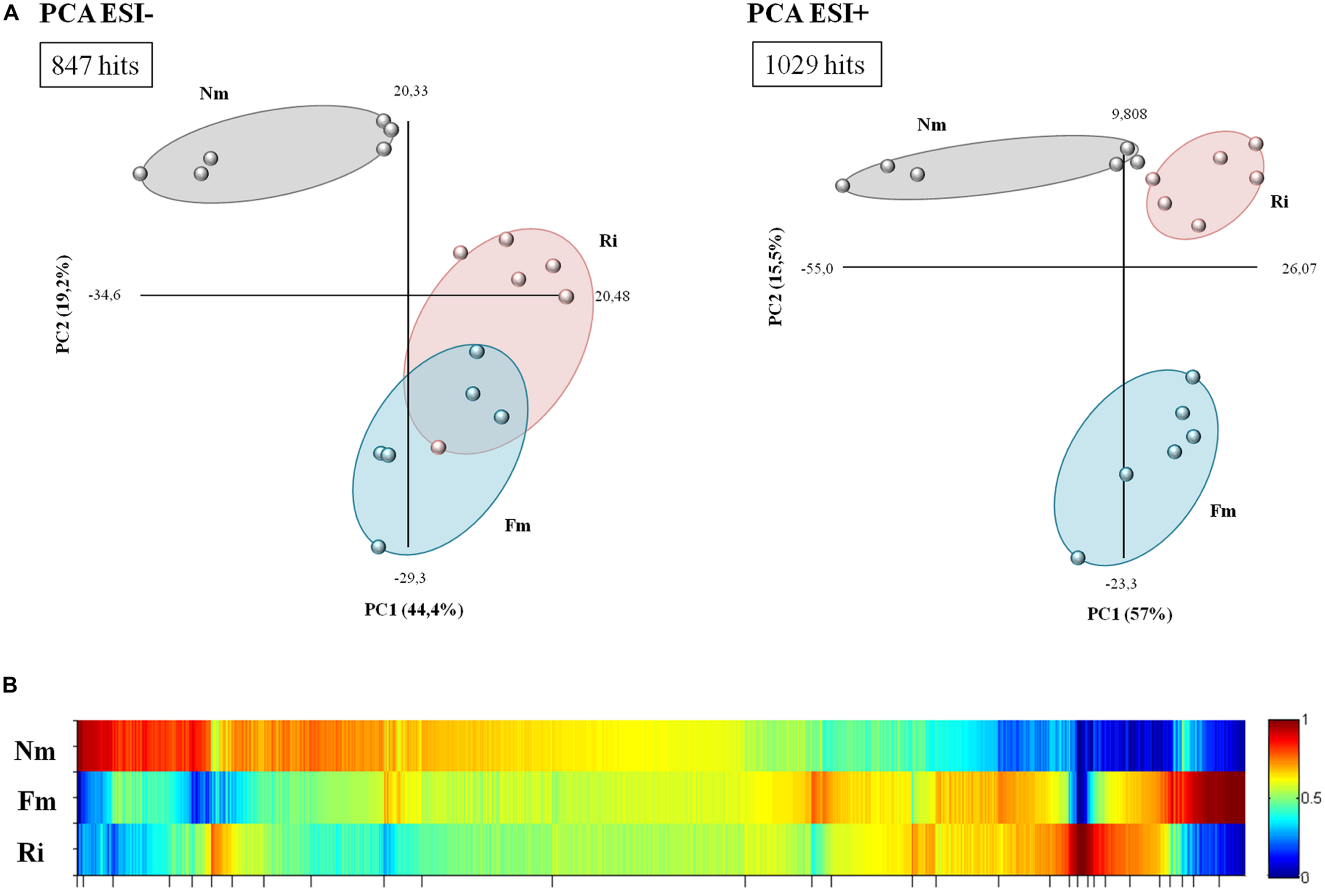
**Overview of metabolite behavior in non-mycorrhizal (Nm) and mycorrhizal roots interpreted using principal component and heat map analysis. (A)** Analysis of major sources of variability of ESI- and ESI+ signals obtained from a non-targeted analysis by HPLC–QTOF MS monitoring metabolomic changes in roots colonized by *F. mosseae* (Fm) or *R. Irregularis* (Ri). Data points represent six replicates per treatment injected randomly into the HPLC–QTOF MS. The identified signals corresponding to different treatments were compared using the non-parametric Kruskal–Wallis test, and only data with a *p* < 0.1 between groups were used for a supervised analysis. **(B)** Heat map of the metabolite profiling, generated with MarVis Filter and Cluster packages, following a Kruskal–Wallys test (*p* < 0.05) by combining positive and negative electrospray ionization analysis. Each color band represents a single compound detected in Nm, Fm, and Ri, whose accumulation is indicated for each treatment by the indicated color scale ranging from high (red) to low (blue) accumulation. The concentration of the metabolites was determined in all samples by normalizing the chromatographic pick area for each compound with the dry weight of the corresponding sample.

Untargeted metabolomic analysis of root extracts via HPLC coupled with a quadrupole time-of-flight mass spectrometer revealed a total of 1407 signals in ESI- mode and 1860 signals in ESI+ mode. A supervised principal component analysis of these signals (*p* < 0.1, 847 and 1029 signals in ESI- and ESI+ mode, respectively) showed a clearly separated behavior between roots colonized by *F. mosseae* (Fm) or by *R. irregularis* (Ri) and non-mycorrhizal roots (Nm), (**Figure 2A**). According to the two main components, no overlap was observed between the mycorrhizal and Nm groups in any of the ESI modes. It is noteworthy that ESI- showed a similar behavior between signals detected for Ri and Fm. Hierarchical cluster analysis of the different groups confirmed previous observations: signals from roots colonized by both AMF clustered closely compared to those from Nm roots (Supplementary Figure S1A).

The clusters corresponding to compounds with the most contrasting accumulation patterns across different treatments were selected from the heat map analysis (**Figure 2B**) for detailed analysis. The clusters of selected signals were analyzed separately (Supplementary Table S2). Firstly, the number of over-accumulated compounds specific to Nm, Fm, and Ri was subtracted from the heat map. Secondly, those signals that were highly accumulated in two of the experimental conditions (such as Nm + Fm, Nm + Ri, or Fm + Ri) were also isolated for subsequent Venn-diagram and pathway analysis (Supplementary Figure S1B and Table S2). The selection, including 1876 signals, contained 300 differentially accumulated in mycorrhizal roots, as illustrated in the Venn diagram (Supplementary Figure S1B). These signals are of interest as they may correspond to compounds relevant for the known benefits of the mycorrhizal interaction, including improved host stress resistance. These metabolites can be generated as a plant response to the AMF colonization or by the AMFs themselves. Despite the core of compounds highly accumulated in both AM roots, there are many specific signals only triggered either by Fm (85 signals) or Ri (35 signals), (Supplementary Figure S1B). Interestingly, the metabolic impact of Fm is stronger than that of Ri. These results suggest that tomato plants retain common responses to different AMF, as they are conserved in both interactions, but there is, in addition, a set of responses that may be specific to particular interactions.

In order to understand the biological meaning of this metabolic transition, we classified the signals contained in the selected clusters from the heat map analysis and Venn diagram (Supplementary Figure S1B) into a pathway ontology using the MarVis Pathway 2.0 (Kaever et al., 2014) linked to the KEGG *Solanum lycopersicum* database (Supplementary Table S2). We particularly focused on those signals that were strongly reduced in both mycorrhizal treatments (Cluster 1), signals highly accumulated in both mycorrhizal interactions (Cluster 2), signals exclusively accumulated in Fm colonized roots (Cluster 3), and finally, signals exclusively accumulated in Ri colonized roots (Cluster 4). Clearly, the major impact of AM on plant metabolism takes place in the primary metabolism, mainly in the amino acid and sugar metabolism (including many hits among the tricarboxylic and other carboxylic acids) but also in some specific secondary metabolites, such as phenolic alcohol derivatives, vitamins, and plant hormones, particularly oxylipins and cytokinins (Supplementary Table S2). Both fungi impacted the 13-LOX oxylipin pathway, with multiple hits in the linoleic and α-linolenic acid metabolism. Remarkably, a clearly overrepresented category in mycorrhiza-enriched compounds is that of metabolites related to ATP-binding cassette (ABC) transporters. ABC transporters are largely expressed in roots and mediate the transport of many secondary metabolites with signaling and defensive functions (Yazaki, 2006).

A closer look at the pathways containing signals with lower levels in mycorrhizal roots (Cluster 1) revealed that other compounds from the same pathways are strongly over-represented in AM. This suggests that the reduction of these compounds in mycorrhizal roots is a consequence of the metabolic flux along the pathways that reduces substrates of a given reaction accumulating the product compounds. Regarding the specific signals, Fm had a stronger impact on amino acids, sugars, and phenolics compared with Ri.

### Impact of the Arbuscular Mycorrhizal Symbiosis on Amino Acid Metabolism

**FIGURE 3:**
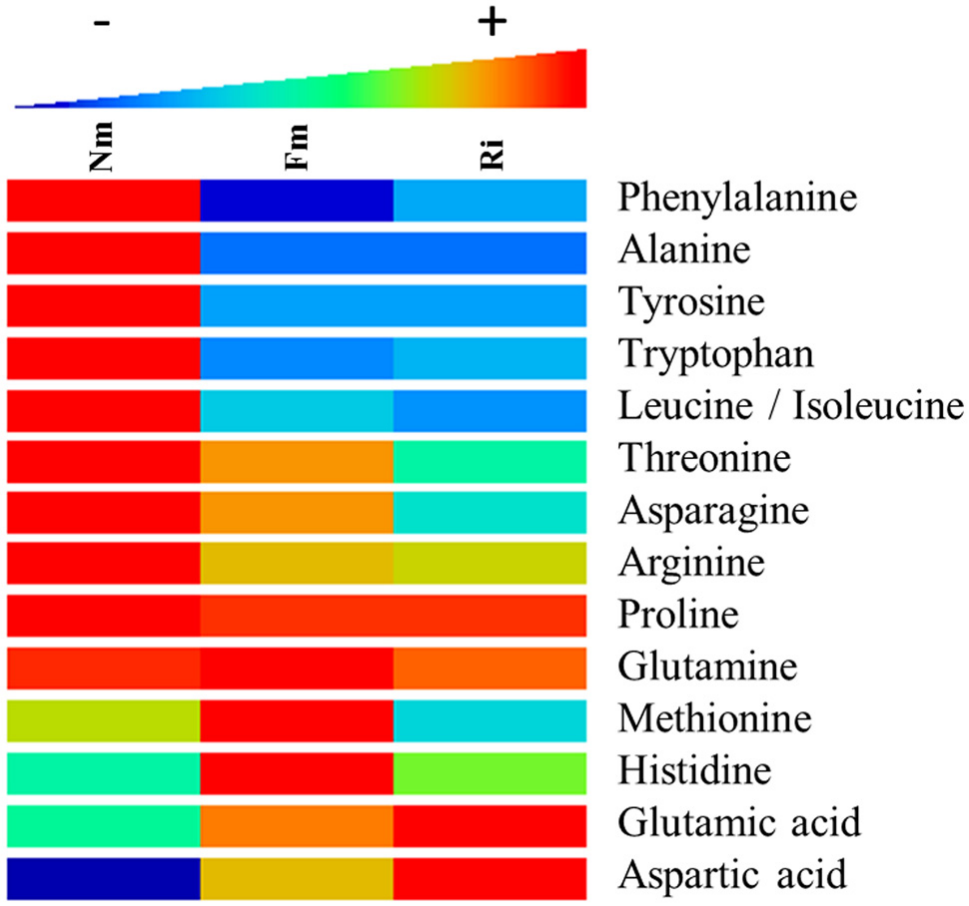
**Heat map analysis of amino acid content in non-mycorrhizal (Nm), *F. mosseae*-colonized (Fm) and *R. irregularis*-colonized roots (Ri).** Samples for analysis were collected 8 weeks after fungal inoculation. Data points represent six biological replicates injected randomly into the HPLC–QTOF MS. Color scale represents the variation in the accumulation of the amino acids, from high (red) to low (blue) contents. Signals corresponding to different treatments were compared using the non-parametric Kruskal–Wallis test, only data with a *p* < 0.1 (between groups) were used for a supervised analysis. Values are relative to the sample dry weight and normalized to the lowest amount.

One of the major pathways altered in mycorrhizal roots was the metabolism of the amino acids (**Figure 3** and Supplementary Table S2). Phe, Tyr, Tryp, and Leu/Ile were consistently less concentrated in mycorrhizal roots colonized by both AMF. Contrastingly, many metabolites derived from Cys, Lys, Ala, Gln, Phe, Tyr, and Trp were highly accumulated in mycorrhizal roots (Supplementary Table S2). The higher concentration of amino acid derived metabolites in AM would explain the lower concentration of free amino acids as metabolic sources (**Figure 3**). It is noteworthy that Phe and Tyr are the main amino acids that generate phenolic acids and their derivatives, highly accumulated in AM roots. This observation suggests a very likely circulation of the basic amino acids into more complex secondary metabolites, which are indeed found in higher concentrations in the symbiotic roots. On the other hand, glutamate (Glu) and aspartate (Asp) were found in higher levels in both mycorrhizal roots. As their active role in the incorporation of N in AM plants is well reported (Govindarajulu et al., 2005; Schliemann et al., 2008), their higher levels are consistent with the increase in N observed in both mycorrhizal treatments (**Figure 1B**). Finally, some AMF-dependent regulation of the amino acids was also observed. For example, His and Met accumulated at higher levels in Fm, whilst they were hardly present in Nm and Ri.

### Impact of the Arbuscular Mycorrhizal Symbiosis on Amino Acid Derived Compounds: Phenolic Alcohol Derivatives, Benzylisoquinolines, and Conjugated Polyamines

**FIGURE 4:**
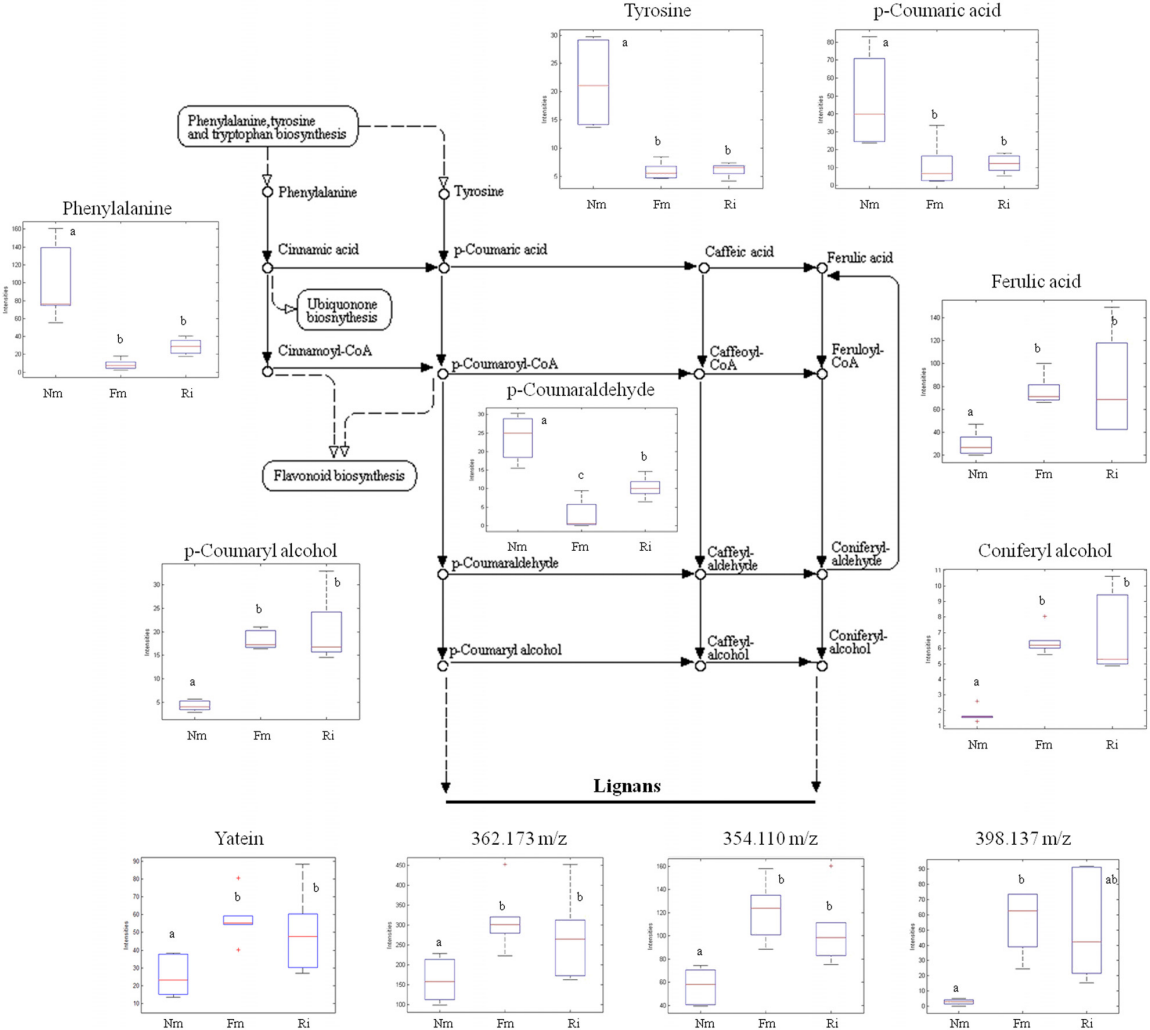
**Profile of selected metabolites from the phenylpropanoid, lignin, and lignan biosynthetic pathways.** Non-mycorrhizal roots (Nm), and roots colonized by *F. mosseae* (Fm) and *R. irregularis* (Ri) were processed for relative quantification analysis by HPLC–QTOF MS. The metabolite concentration in each sample was determined by normalizing the chromatographic area for each compound with the dry weight of the corresponding sample. For those compounds matching two or more identification criteria (exact mass, positive fragmentation spectrum, or chemical standards) names are assigned. Those compounds only tentatively identified are assigned only by an m/z ratio. Dotted arrows mean multiple metabolic steps, straight arrows mean single steps. Data in the same plot not sharing a common letter differ significantly (*p* < 0.05) according to the Newman–Keuls test.

The phenyl-alcohol metabolism was also strongly affected in AM. This metabolism includes deamination of Phe and Tyr by the Phenylalanine ammonia lyase enzyme (Cochrane et al., 2004), after which a set of phenolic acids are converted into aldehydes and alcohols by successive reductions. The phenolic alcohols are precursors of important cell wall components such as monolignans and lignins. In addition, coumaryl and coniferyl alcohols can be converted into more complex flavonoids with cell protective functions (Wang et al., 2013). As described above, the identified upstream compounds of this pathway, Phe and Tyr, were found in lower levels in mycorrhizal roots (**Figures 3** and **4**), while the content of other intermediary compounds, such as ferulic acid, cumaryl alcohol, and coniferyl alcohol, was higher in the colonized roots (**Figure 4**). Additionally, other tentatively identified monolignans (400.152, 362.173, 354.110, and 398.137 m/z) were also more concentrated in mycorrhizal roots (**Figure 4**). These observations suggest that AMF stimulates a reorganization of specific cell wall components.

Other amino acid derived compounds related to defense were also found in higher levels in mycorrhizal roots. Plants often produce alkaloids to defend themselves against pests, diseases, and other external biological stimuli. Several mass signals identified as benzylisoquinoline alkaloids (BIAs), such as 271.107; 369.126; 332.112; and 273.124 m/z, were found in high quantities in AMF colonized roots (Supplementary Figure S2). Higher accumulation of several polyamines and their conjugates were also found, some putatively identified as spermidine (145.112 m/z), tricaffeoyl spermidine (631.274 m/z), and triferuloyl spermidine (673.242 m/z), (Supplementary Figure S2).

### Impact of Arbuscular Mycorrhizal Symbiosis on the Oxylipin Pathway

**FIGURE 5:**
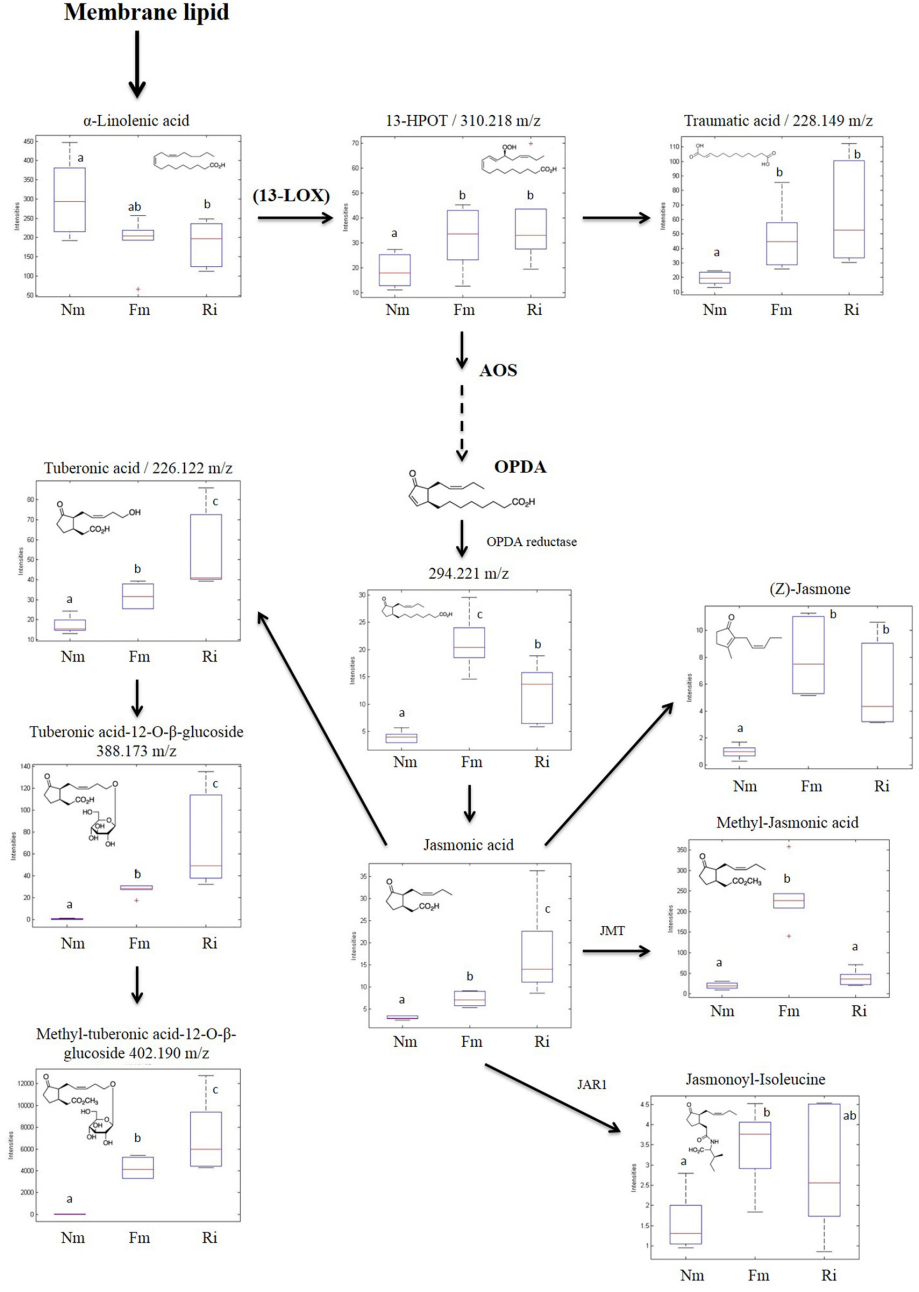
**Accumulation profile of compounds related to the 13-LOX oxylipin pathway.** Non-mycorrhizal (Nm), *F. mosseae* (Fm) and *R. irregularis* (Ri) colonized roots were processed for relative quantification analysis by HPLC–QTOF MS. Concentration of metabolites was determined in all the samples by normalizing the chromatographic area for each compound with the dry weight of the corresponding sample. For those compounds matching two or more identification criteria (exact mass, positive fragmentation spectrum, or chemical standards) names are assigned. Those compounds tentatively identified are assigned by an m/z ratio together with their putative names. Dotted arrows mean multiple metabolic steps, straight arrows mean single steps. Data in the same plot not sharing a common letter differ significantly (*p* < 0.05) according to the Newman–Keuls test.

The untargeted metabolomic analysis revealed α-linolenic acid derivatives as major metabolic targets for mycorrhizal symbiosis. Among the 45 signals related to the oxylipin pathways altered in mycorrhizal roots (Supplementary Table S2), 11 compounds were fully identified by either exact mass or fragmentation spectrum (**Figure 5**), and they corresponded to the 13-LOX branch of the oxylipin pathway. This branch leads to the biosynthesis of the phytohormone JA and derivatives, known to be altered in AM in different plant species (Wasternack and Hause, 2013; Fernández et al., 2014). With the exception of α-linolenic acid, the source metabolite, the compounds identified in this pathway showed higher levels in mycorrhizal roots (**Figure 5**). Most of them showed higher concentrations in roots colonized by both AMF, although to different levels depending on the particular compound and the colonizing fungi. Remarkably, the levels of the bioactive forms of JA methyl-JA (Me-JA) and JA-Ile conjugates were accumulated in significantly higher levels only in the Fm roots.

## Discussion

Several studies have detailed the transcriptional reprogramming in the host plant during interaction with AMF, not only transiently during the establishment of the symbiosis, but also in the maintenance of the symbiosis (Hohnjec et al., 2005; Liu et al., 2007; Guether et al., 2009). While there is evidence for these mycorrhiza associated transcriptional changes in multiple plant families, the metabolic impact on host roots have been only monitored in a few plant species, mainly legumes (Schliemann et al., 2008; Laparre et al., 2014). Our research presents a complete metabolomic analysis in roots of a relevant crop, tomato, in symbiotic interaction with two different AMF: *F. mosseae* and *R. irregularis*, both known to increase tomato resistance against biotic and abiotic stresses and yield (Pozo et al., 2002; Fritz et al., 2006; Aroca et al., 2008; Gianinazzi et al., 2010; Barzana et al., 2012; Vos et al., 2012). These fungi are among the most studied and widely distributed AMF in agricultural and ecological settings. *R. irregularis* is the most commonly used AMF in commercial inoculants, and is widely used as a model organism in AM research, because it is readily grown using *in vitro* cultivation in monoxenic conditions, and its genome is now available (Declerck et al., 2005; Tisserant et al., 2013). In contrast, *F. mosseae* cannot be cultivated in monoxenic cultures but is usually very efficient in increasing host resistance to pests and pathogens (Jung et al., 2012).

Increases in P levels in the host following root colonization by AMF is one of the major and most reported benefits of mycorrhizal interactions, although the increase depends on the partners involved and the experimental conditions (Smith and Smith, 2015). However, AM are not always associated to increased vegetative biomass (Smith and Smith, 2011), and improved stress tolerance has been proposed as another major benefit of the symbiosis (Gianinazzi et al., 2010; Selosse et al., 2014). In our experimental system, both *F. mosseae* and *R. irregularis* colonization increased the total P levels in tomato roots (cv. Moneymaker), whilst shoot and root biomass, root/shoot ratio and total carbon content were not significantly altered. Interestingly, total N was significantly higher in mycorrhizal tomato roots, and consequently the C/N ratio was reduced.

Our untargeted metabolome analysis confirmed, following restrictive statistical analyses, that the metabolome of mycorrhizal tomato roots is significantly different from that of non-mycorrhizal tomatoes. Pioneering work by Schliemann et al. (2008), and later Laparre et al. (2014) showed that there are clear differences in the development and symbiosis-dependent primary and secondary metabolism of *M. truncatula* roots colonized by the AMF *R. irregularis*.

A large number of signals related to sugar and carboxylic acid metabolism showed elevated levels in mycorrhizal roots. This suggests that primary sugar metabolism was activated by the symbiosis as has been shown in multiple mycorrhizal systems (Bago et al., 2000; Zouari et al., 2014) probably related to an increase in the host photosynthesis to increase C-fixation (Kaschuk et al., 2009). Total C content, however, remained unaltered in mycorrhizal roots, probably due to the fact that part of the host-derived C is taken by the fungal partner to maintain the mycelial network (Bago et al., 2000). Indeed, transcriptional regulation of carbohydrate related genes and activation of sugar transporters are reported in AM (Bago et al., 2000; Doidy et al., 2012). Another notable target of mycorrhizal metabolic reprogramming corresponds to amino acid metabolism, which is one of the pathways with more hits amongst the AM-related differential signals (Supplementary Table S2). A strong reduction in the accumulation of several amino acids (Trp, Tyr, Phe, Ala, Leu) was observed, probably because of their function as source compounds of amino acid-derived secondary metabolites (**Figure 3**). For example, Phe and Tyr are precursors of the phenylpropanoid pathway, and several intermediaries of this pathway were found to be highly accumulated in the mycorrhizal roots. Both amino acids were found in low levels in mycorrhizal tomato roots, and the data suggest that most phenolic derivatives may have been redirected to the formation of lignans and lignins. An increase in lignans has been previously reported in other root-beneficial fungus interaction involving *Piriformospora indica* (Baldi et al., 2010). Regarding the lignins, it has been shown that mycorrhizal colonization can increase the lignin content of the root cell walls (Ziedan et al., 2011), and cell-wall lignification is one of the proposed mechanisms restricting penetration by phytopathogenic fungi in mycorrhizal roots (Jung et al., 2012). Remarkably, a similar reduction of amino acid content was observed in aboveground tissues of mycorrhizal *L. japonica* (Fester et al., 2011), and *Arabidopsis* plants treated with the defense-priming agent β-amino butyric acid showed a lower content of all amino acids except Glu (Pastor et al., 2014). Thus, it is tempting to speculate that the reduction of basic and aromatic amino acids is a common response to defense-priming stimuli. However, this putative relationship requires further experimental confirmation.

Despite an overall reduction in most amino acids, our study revealed a higher accumulation in the mycorrhizal roots of Glu and Asp (**Figure 3**), important amino acids in uptake of N by AMF extra-radical mycelium (Govindarajulu et al., 2005). The elevated N levels observed in AM, together with the elevated levels of these amino acids, also reported in other mycorrhizal systems (Schliemann et al., 2008) suggest that N uptake and assimilation is stimulated in mycorrhizal tomato roots. An impact of AMF colonization on enzymes catalyzing the biosynthesis of N rich compounds such as alkaloids has been also described (Zeng et al., 2013). Our metabolomic analysis showed that both BIAs and conjugated polyamines, all with defense-related functions in plants, are also over accumulated in mycorrhizal compared to non-mycorrhizal roots.

One of the clear targets of root reprogramming in mycorrhizal roots was the oxylipin pathway. All the metabolites of the 13-LOX branch identified either through the fragmentation spectrum, exact mass or using standards, were more concentrated in mycorrhizal roots, including several bioactive forms of the phytohormone JA. This may explain the reduced levels of the source metabolite α-linolenic acid in AM. The induction of most of the intermediates of the pathway is consistently reproduced in both mycorrhizal root systems, although the relative levels of the different compounds differ according to the AMF. The alteration of multiple metabolites in the pathway, known to be precisely regulated by inter conversion among them (Wasternack and Hause, 2013) and differential accumulation in response to the particular AMF, support their involvement in fine-tuning of metabolic reprogramming in response to particular growth conditions, the partners involved and the symbiotic stage reached (Wasternack and Hause, 2013; Fernández et al., 2014). Elevated levels of the JA-related volatile compounds Me-JA and CIS-jasmone, both with known roles in defense against biotic stresses, are reported here for the first time in mycorrhizal roots. Moreover, Me-JA is also known to be involved in the plant response to abiotic stresses like drought or salinity (Fahad et al., 2015). The exact role of JA and its derivatives in the control of AM remains controversial, as exogenous application of the hormone provides contrasting results, and JA deficient mutants have relatively subtle mycorrhizal phenotypes that seem to depend largely on the host plant species (Wasternack and Hause, 2013). In some plant systems LOX-silencing does not significantly affect AMF colonization, therefore it has been proposed that activation of JA-signaling is a downstream event triggered by this symbiosis (Wasternack and Hause, 2013). In fact, Pv*LOX2-*silencing in common bean roots is reported to have no effect on mycorrhiza establishment, whilst it does impair mycorrhiza-induced resistance (Mora-Romero et al., 2014). Thus, the results suggest that AM functioning implies a precise regulation of the oxylipin pathway that may contribute to improving stress resistance in mycorrhizal plants. It is noteworthy that the genes coding for alkaloid biosynthetic enzymes are JA-inducible (Mishra et al., 2013; Wasternack and Hause, 2013). Additionally, phenylpropanoid-polyamine conjugated (PPCs), other N rich compounds related to defense responses, were found in elevated levels in the mycorrhizal roots and are also described to be under JA regulation in several plants, including tomato (Kaur et al., 2010).

Although the metabolic pathways altered by the mycorrhizal symbiosis were common to both *F. mosseae* and *R. irregularis* interactions, many compounds showed specific responses to one of the interactions. These results suggest, therefore, a fine-tuned developmental regulation of these pathways in an AMF-dependent manner. In terms of fitness costs, we have observed no reduction in plant biomass despite the extensive metabolic changes encountered in AM roots. AMF-induced resistance against soil pathogens in tomato roots seems to be related to cost-efficient defense regulation mechanisms (Pozo et al., 2002; Steinkellner et al., 2012; Vos et al., 2012; Selosse et al., 2014). There is experimental evidence of the role of the JA-signaling pathway in priming of plant defenses by mycorrhizas (Song et al., 2013). The contribution of other particular metabolic pathways altered in mycorrhizal roots to the enhanced host stress resistance–tolerance remains to be experimentally demonstrated.

To sum up, following a non-targeted full metabolomic approach, the metabolic transition in roots colonized by two widely distributed AMF, *F. mosseae* and *R. irregularis*, was successfully characterized. An important reprogramming of some major metabolic pathways in both mycorrhizal interactions was observed, pointing to common responses associated to AM, although there were also some AMF-specific responses. Many of the changes are related to plant defense mechanisms, and may underlay the well-known effects of the interaction on plant-stress tolerance. The identification of differentially regulated pathways in this study is instrumental to functional studies aiming to reveal the mechanistic basis of AM benefits, such as the improved resistance–tolerance to biotic and abiotic stresses. Moreover, these studies can pave the way to improving the biotechnological applications of AMF in agricultural settings and in the production of plant secondary metabolites with medicinal or nutritional properties (Gianinazzi et al., 2010; Pedone-Bonfim et al., 2015).

## Conflict of Interest Statement

The authors declare that the research was conducted in the absence of any commercial or financial relationships that could be construed as a potential conflict of interest.

## Footnotes

1 http://www.i-beg.eu

2 http://www.r-project.org/

3 http://www.numericaldynamics.com/
