# Supplementary material for: Metabolic transition in mycorrhizal tomato roots
Source: Front Microbiol. 2015 Jun 23;6:598. doi: 10.3389/fmicb.2015.00598 (PMC4477175; doi:10.3389/fmicb.2015.00598)

SUPPLEMENTARY FIGURE 1. **A)** Hierarchical clustering of both ESI+ and ESI- compounds found in non-mycorrhizal (Nm), *F. mosseae* (Fm) and *R. irregularis* (Ri) colonized roots. Samples were collected 8 weeks after inoculation with Fm or Ri. For each treatment, six replicates were injected randomly into the HPLC-QTOF-MS. The signals corresponding to different treatments were compared using the non-parametric Kruskal-Wallis test, and only data with a $p<0.1$ between groups was used for a supervised analysis. The clustering was performed using the package MarVis Filter from the software MarVis 2.0. **B)** Venn diagram of combined ESI+ and ESI- compounds obtained from the selected clusters represented in the Figure 2 and table S2. Numbers inside the shared coloured region represents the number of shared differentially accumulated compounds either by Nm-Fm, Nm-Ri or Fm-Ri.

A)

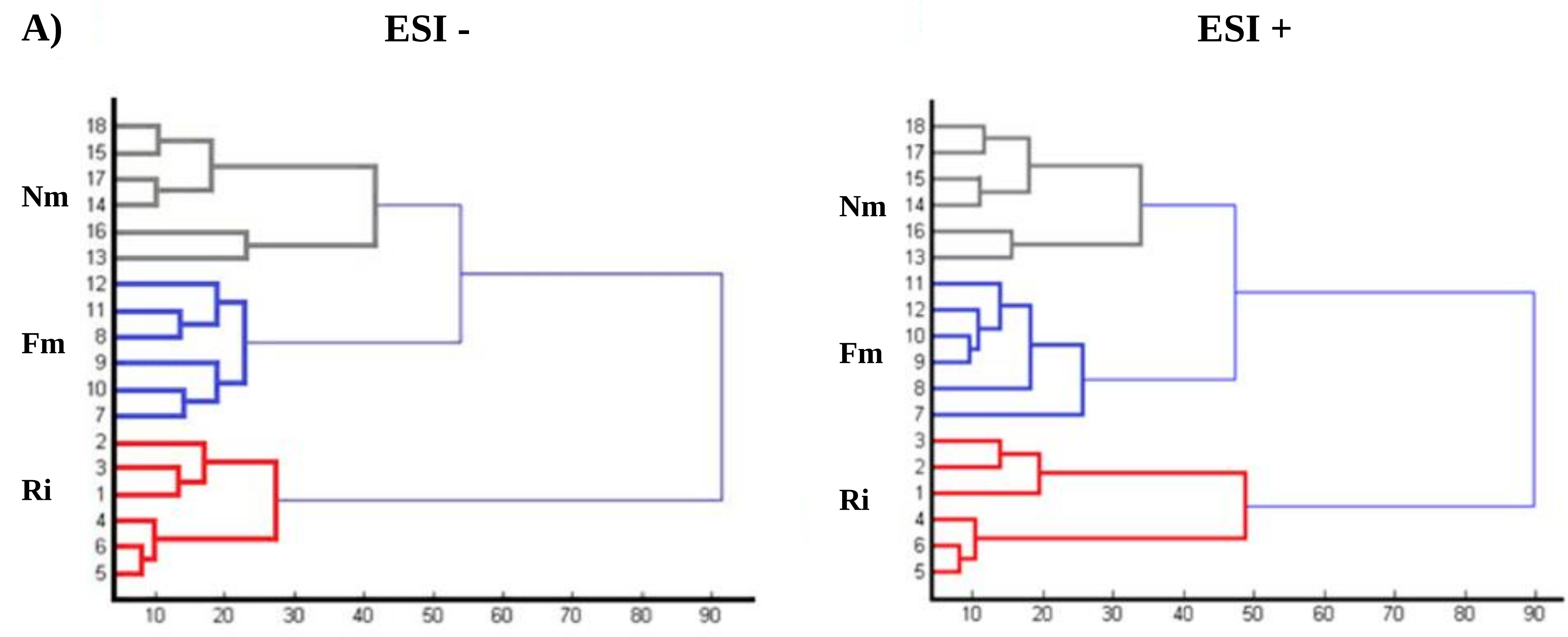

B)

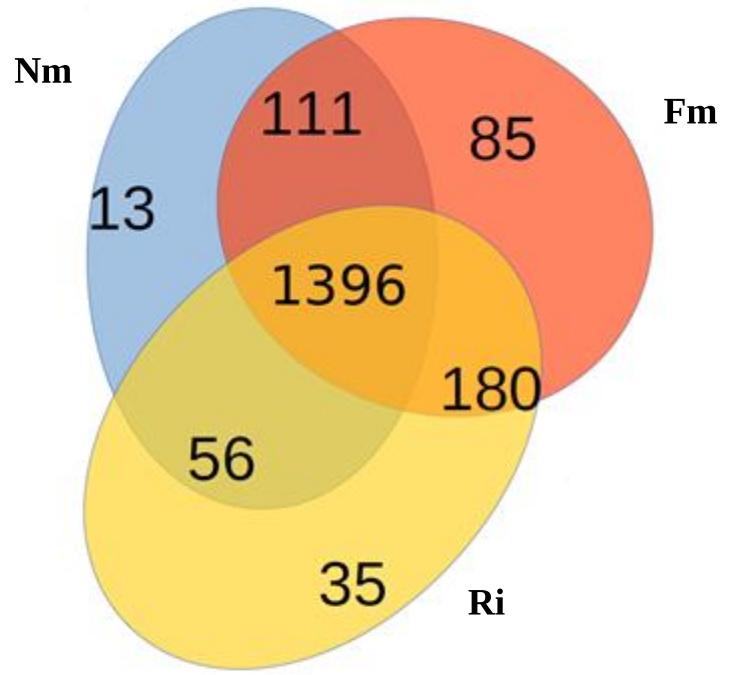

Supplement: Supplementary file 1 [file Presentation_1.PDF]
