# Supplementary material for: Metabolic transition in mycorrhizal tomato roots
Source: Front Microbiol. 2015 Jun 23;6:598. doi: 10.3389/fmicb.2015.00598 (PMC4477175; doi:10.3389/fmicb.2015.00598)

SUPPLEMENTARY FIGURE 2. Polyamine phenylpropanoid conjugates (PPCs) and benzylisoquinoline alkaloids (BIAs) differentially accumulated in non-mycorrhizal (Nm), *F. mosseae*-(Fm) and *R. irregularis*-(Ri) colonized roots. For each treatment, six replicates were injected randomly into the HPLC-QTOF MS. Compounds tentatively identified were assigned by a m/z ratio together with their putative names.

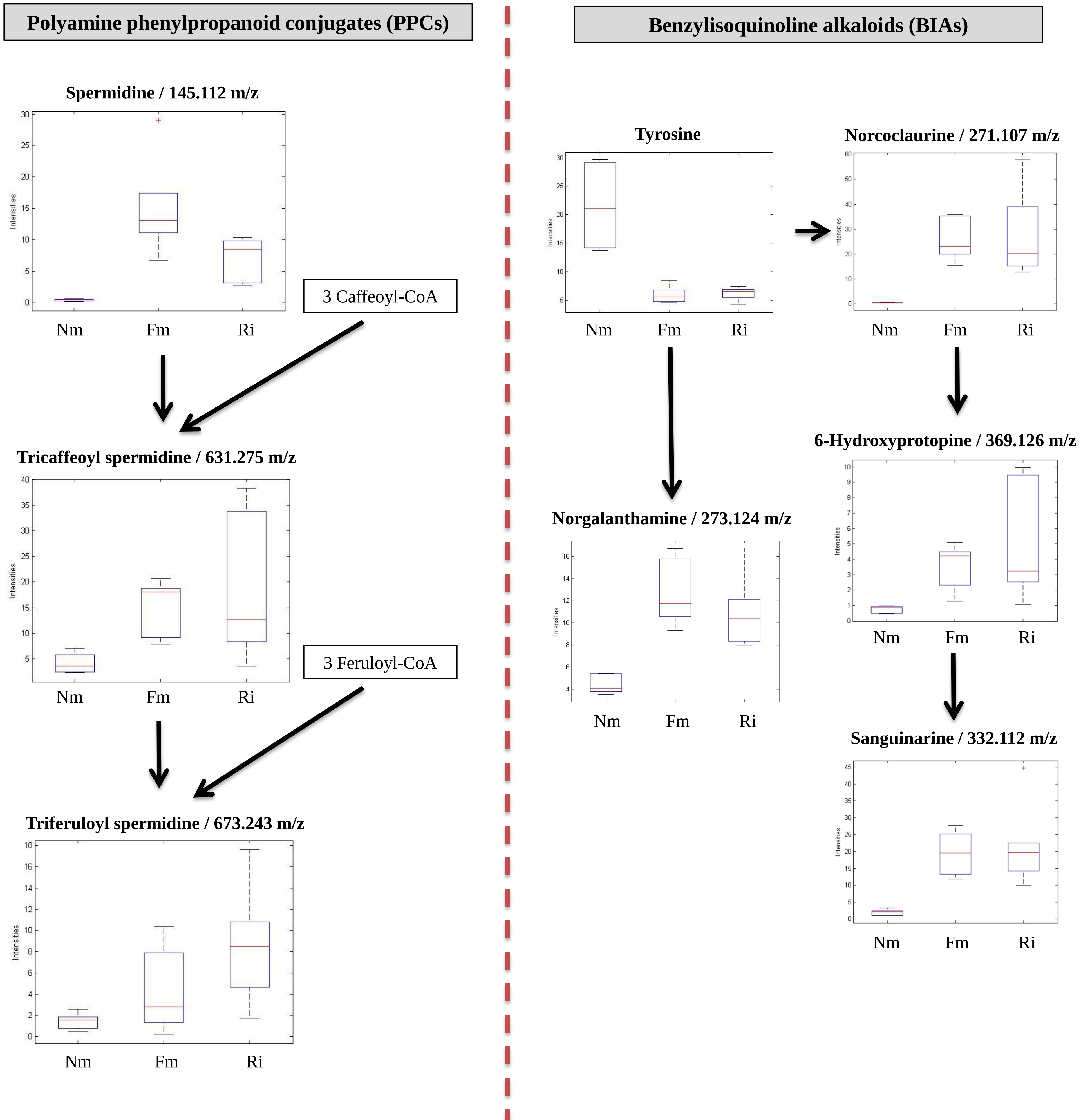

Supplement: Supplementary file 2 [file Presentation_2.PDF]
