# Supplementary material for: Metabolic transition in mycorrhizal tomato roots
Source: Front Microbiol. 2015 Jun 23;6:598. doi: 10.3389/fmicb.2015.00598 (PMC4477175; doi:10.3389/fmicb.2015.00598)
Supplement: Supplementary file 3 [file Presentation_3.PDF]

SUPPLEMENTARY TABLE 1. Shoot and root fresh weight, nitrogen (N) and carbon (C) content, and C/N ratio of non-mycorrhizal (Nm) and mycorrhizal tomato plants eight weeks after inoculation with *F. mosseae* (Fm) or *R. irregularis* (Ri). Fresh weight data represent the means of ten independent biological replicates ± SE. P, N and C content and C/N ratio represent the means of three independent biological replicates each consisting of a pool of roots from three independent plants ± SE. Data in the same column not sharing a common letter differ significantly ($p<0.05$) according to Student-Newman-Keuls test.

Treatment	FW Shoot (g)	FW Root (g)	Root : Shoot	Ntotal (g/100g)	Ctotal (g/100g)	C/N
**Nm**	10,27 ± 1,57 a	4,68 ± 0,66 a	0,48 ± 0,03 a	1,91 ± 0,06 a	32,69 ± 0,35 a	17,17 ± 0,45 a
**Fm**	11,80 ± 0,54 a	6,42 ± 0,43 a	0,54 ± 0,02 a	2,14 ± 0,03 b	30,50 ± 1,21 a	14,26 ± 0,47 b
**Ri**	10,56 ± 0,62 a	5,53 ± 0,48 a	0,52 ± 0,02 a	2,23 ± 0,22 ab	31,75 ± 2,82 a	14,29 ± 0,58 b
