# Supplementary material for: Metabolic transition in mycorrhizal tomato roots
Source: Front Microbiol. 2015 Jun 23;6:598. doi: 10.3389/fmicb.2015.00598 (PMC4477175; doi:10.3389/fmicb.2015.00598)
Supplement: Supplementary file 4 [file Presentation_4.PDF]

SUPPLEMENTARY TABLE 2. Identified pathways and compounds found in selected clusters.

Signals corresponding to different treatments were compared using the non-parametric Kruskal-Wallis test, and only data with a $p<0.1$ between groups were used for a supervised analysis. Quantitative values are relative to the sample dry weight and normalized to the lowest amount, and are represented following a colour scale ranging from blue (low) to red (high) accumulation. The signals have been selected following a criteria of maximum colour differences between treatments. Cluster 1 includes signals from the heatmap overrepresented in non-mycorrhizal roots (Nm), Cluster 2 includes signals from the heatmap overrepresented at the same time in both *F. mosseae* (Fm) and *R. irregularis* (Ri) colonized roots, Cluster 3 includes signals from the heatmap overrepresented only in Fm colonized roots and Cluster 4 includes signals from the heatmap overrepresented only in Ri colonized roots. The table was built grouping the number of hits provided by the package MarVis Pathway (MarVis 2.0) that were organized by pathways inside each cluster.

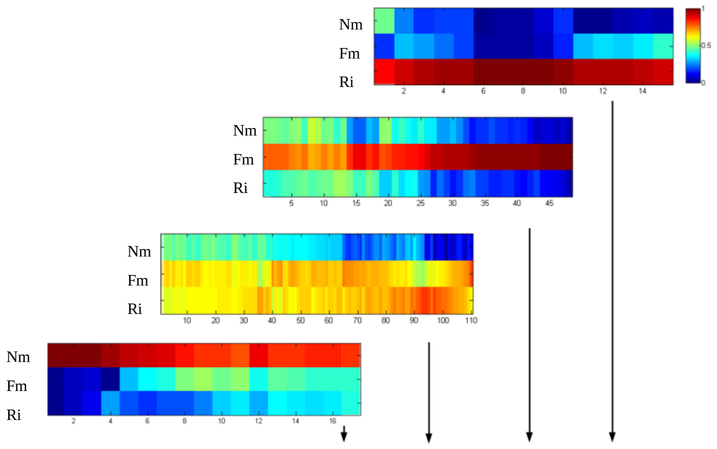

	Solanum lycopersicum (tomato)	CLUSTER 1	CLUSTER 2	CLUSTER 3	CLUSTER 4
	ABC transporters -	-	14	3	1
Aminoacid metabolism	Alanine; aspartate and glutamate metabolism -	-	7	1	2
	Amino acids	-	3	1	1
	Amino sugar and nucleotide sugar metabolism -	-	10	5	1
	Biosynthesis of amino acids -	-	16	9	2
	Cysteine and methionine metabolism -	-	10	1	1
	Aminoacyl-tRNA biosynthesis -	-	3	1	1
	Lysine biosynthesis -	-	4	1	1
	Lysine degradation -	-	4	4	-
	Glycine; serine and threonine metabolism -	-	8	3	2
	Histidine metabolism -	-	5	6	1
	Tryptophan metabolism -	-	12	8	5
	Tyrosine metabolism -	-	7	3	1
	Valine; leucine and isoleucine biosynthesis -	-	5	3	-
	Valine; leucine and isoleucine degradation -	-	2	4	-
	Phenylalanine metabolism -	-	8	-	-
	beta-Alanine metabolism -	-	6	2	1
	Arginine and proline metabolism -	-	12	5	1
Oxylipins	Linoleic acid metabolism -	-	2	-	16
	alpha-Linolenic acid metabolism -	-	9	1	-
	Fatty acid degradation -	-	2	-	-
	Arachidonic acid metabolism -	-	14	-	1
Sugar metabolism	Carbon metabolism -	-	9	6	1
	Citrate cycle (TCA cycle) -	-	4	-	-
	Fructose and mannose metabolism -	-	6	-	-
	2-Oxocarboxylic acid metabolism -	-	21	8	6
	Galactose metabolism -	-	8	1	-
	Pentose and glucuronate interconversions -	-	14	6	-
	Pentose phosphate pathway -	-	5	4	-
	Glycolysis / Gluconeogenesis -	-	3	-	-
	Pyruvate metabolism -	-	6	-	-
	Starch and sucrose metabolism -	-	6	-	-
	Glyoxylate and dicarboxylate metabolism -	-	10	3	-
Phenolic alcohols and derivatives	Isoquinoline alkaloid biosynthesis -	2	17	4	-
	Lignans	-	14	4	1
	Phenylpropanoid biosynthesis -	-	7	2	1
	Flavone and flavonol biosynthesis -	-	3	1	-
	Flavonoid biosynthesis -	-	5	1	2
	Stilbenoid; diarylheptanoid and gingerol biosynthesis	-	3	2	2
	Limonene and pinene degradation -	-	16		
	Monoterpenoid biosynthesis -	-	14	2	7
	Ubiquinone and other terpenoid-quinone biosynthesis -	-	6	2	1
	Diterpenoid biosynthesis -	6	8	3	-
	Purine metabolism -	-	12	5	2
	Pyrimidine metabolism -	-	12	6	-
	Nicotinate and nicotinamide metabolism -	-	5	3	1
	Tropane; piperidine and pyridine alkaloid biosynthesis -	-	10	4	1
Vitamins	Thiamine metabolism -	-	3	-	-
	Riboflavin metabolism -	-	3	2	-
	Vitamin B6 metabolism -	-	7	1	-
	Pantothenate and CoA biosynthesis -	1	8	2	1
	Folate biosynthesis -	1	4	1	4
	Plant hormone signal transduction -	-	2	-	-
	Zeatin biosynthesis -	-	6	1	1
	Porphyrin and chlorophyll metabolism -	-	9	2	-
	Propanoate metabolism -	-	3	1	-
	Butanoate metabolism -	-	6	2	-
	Carotenoid biosynthesis -	1	2	-	-
	Glucosinolate biosynthesis -	-	10	6	5
	Glutathione metabolism -	-	3	-	-
	Glycerolipid metabolism –	-	5	5	-
	Inositol phosphate metabolism -	-	2	-	-
	Ascorbate and aldarate metabolism -	-	7	7	-
