# Supplementary material for: Metabolic transition in mycorrhizal tomato roots
Source: Front Microbiol. 2015 Jun 23;6:598. doi: 10.3389/fmicb.2015.00598 (PMC4477175; doi:10.3389/fmicb.2015.00598)
Supplement: Supplementary file 5 [file Presentation_5.PDF]

SUPPLEMENTARY TABLE 3. HPLC-QTOF-MS library of standards compounds used for identification of metabolites based on exact mass and retention time matching.

**Compound**	**Exact mass**	**Ion mass**	**Ionization mode**	**Retention time (min)**
Salicylic Acid	138,032	137,024	ESI-	12,2
Cinamic Acid	148,052	147,045	ESI-	10,5
Indole acetic acid	175,063	174,055	ESI+/ESI-	6,3
Caffeic Acid	180,042	179,035	ESI-	4,0
Jasmonic Acid	210,126	209,118	ESI-	11,1
Abscisic Acid	264,136	263,128	ESI-	9,6
Jasmonate-Isoleucin	323,210	322,202	ESI-	13,4
Ferulic Acid	194,058	193,050	ESI-	5,6
OPDA	292,204	291,196	ESI-	13,0
SAG	300,085	299,077	ESI-	4,1
SGE	300,085	299,077	ESI-	4,1
Chlorogenic acid	354,095	353,087	ESI-	4,4
ABA-Glucoside	426,189	425,181	ESI-	4,9
Camalexin	200,041	201,049	ESI+	11,8
Glycine	750,320	760,398	ESI+	Not retained
Alanine	890,477	900,555	ESI+	0,8
Arginine	174,112	175,120	ESI+	0,5
Asparagine	132,054	133,061	ESI+	0,8
Aspartic Acid	133,038	132,030	ESI-	1,4
Cysteine	121,020	122,028	ESI+	0,8
Glutamine	146,069	147,077	ESI+	0,8
Glutamic acid	147,053	148,061	ESI+	0,8
Histidine	155,070	156,077	ESI+	0,6
Isoleucine	131,095	132,102	ESI+	1,4
Leucine	131,095	132,102	ESI+	1,4
Lysine	146,106	147,113	ESI+	0,6
Methionine	149,051	150,059	ESI+	1,1
Phenylalanine	165,079	166,087	ESI+	2,1
Proline	115,063	116,071	ESI+	0,8
Serine	105,043	106,050	ESI+	0,8
Threonine	119,058	120,066	ESI+	0,8
Tryptophan	204,090	205,098	ESI+	2,6
Tyrosine	181,074	182,082	ESI+	1,2
Valine	117,079	118,087	ESI+	0,9
5-Hydroxyindole-3-acetic acid	191,058	192,066	ESI+	3,4
Indole-3-acetamide	174,079	175,087	ESI+	4,1

Compound	Exact mass	Ion mass	Ionization mode	Retention time (min)
N-(3-indoleylacetyl)-L-alanine	246,100	247,108	ESI+	8,1
Indole-3-carboxaldehyde	145,053	146,061	ESI+	5,5
Methyl indole-acetate	189,079	190,087	ESI+	10,3
N-[(-)-Jasmonoyl]-Methionine (JAMet)	341,160	340,150	ESI-	12,7
Jasmonoyl-L-phenylalanine	357,194	356,186	ESI-	13,3
Jasmonoyl-L-valine (JAVal)	309,194	308,186	ESI-	12,5
Indole-3-acetyl-Isoleucine	288,147	289,155	ESI+	12,1
Indole-3-acetyl-L-phenylalanine	322,130	323,139	ESI+	12,6
Indole-3-carboxaldehyde	145,053	144,0449/146,0606	ESI-/+ (more neg)	5,3
Indole-3-acetonitrile	156,060	157,077	ESI+	-
Indole-3-acetic acid-L-aspartic acid	290,090	289,082	ESI-	9,8
Indole-3-pyruvic acid	203,058	202,0504/204,0661	ESI-/+	-
I3CA methyl ester	175,060	176,071	ESI+	4,0
Indole-3-acetyl-L-tryptophan	361,142	360,1348/362,1505	ESI-/+	11,9
Adipic Ac	146,141	145,050	ESI -	3,0
Ketoglutaric Ac	146,021	145,013	ESI -	--
Galacturonic Ac,	194,042	193,034	ESI -	1,5
Malic Ac,	134,021	133,014	ESI -	2,1
Folic Ac, (B9)	441,139	440,130	ESI -	4,3?
Fumaric Ac,	116,011	115,016	ESI -	-
Gibberellic Ac,	346,141	345,142	ESI -	5,5
Ascorbic Ac, (C)	176,032	177,060	ESI+	11,5
Maleic Ac,	116,011	115,000	ESI -	-
Nicotinic Ac,	123,032	124,024	ESI+	1,6
p-Aminobenzoic Ac,	137,048	138,050	ESI+	2,7
Pyruvic Ac,	880,160	87,008	ESI -	4,14?
Salicylhydroxamic Ac,	153,043	152,034	ESI -	3,3
Sinapic Ac,	224,069	223,060	ESI -	4,9
Pipecolic Ac,	129,079	128,071	ESI -	0,9
2-Aminoadipic Ac,	161,069	160,061	ESI -	0,9
AMP	347,063	346,050	ESI -	2,6
ADP	427,029	428,030	ESI+	
ATP	506,996	505,987	ESI -	
ATP		508,003	ESI+	
FAD	785,157	786,160	ESI+	-

Compound	Exact mass	Ion mass	Ionization mode	Retention time (min)
NADH	665,120	664,101	ESI -	--
Riboflavin (B2)	376,138	377.146,000	ESI+	3,9
Pyridoxal 5-phosphate (B6)	247,025	248,030	ESI+	4,1
Vanillin	152,047	153,050	ESI+	4,2
Quercetin	302,043	301,030	ESI -	10,9
Naringenin	272,069	271,064	ESI -	10,7
Scopoletin	192,042	193,040	ESI+	4,5
Hesperetin	302,079	303,080	ESI+	10,8
6-Benzylaminopurine	225,101	226,108	ESI+	6,2
Kinetin	215,081	216,080	ESI+	4,2
Thiamine (B1)	265,110	265,110 (siempre cargardo)	ESI+	0,5
Zeatin (not in the library)	219,112	220,119	ESI+	2,44 in real sample
